# Inherited Platelet Disorders: An Updated Overview

**DOI:** 10.3390/ijms22094521

**Published:** 2021-04-26

**Authors:** Verónica Palma-Barqueros, Nuria Revilla, Ana Sánchez, Ana Zamora Cánovas, Agustín Rodriguez-Alén, Ana Marín-Quílez, José Ramón González-Porras, Vicente Vicente, María Luisa Lozano, José María Bastida, José Rivera

**Affiliations:** 1 Servicio de Hematología y Oncología Médica, Hospital Universitario Morales Meseguer, Centro Regional de Hemodonación, Universidad de Murcia, IMIB-Arrixaca, CIBERER-U765, 30008 Murcia, Spain; veronica_93@hotmail.es (V.P.-B.); ana.sanchez1610@gmail.com (A.S.); ana.zamora1@um.es (A.Z.C.); vicente.vicente@carm.es (V.V.); mllozano@um.es (M.L.L.); 2 Servicio de Hematología, Hospital Universitario Ramón y Cajal, 28034 Madrid, Spain; nrecal@gmail.com; 3 Servicio de Hematología, Hospital Virgen de la Salud, Complejo Hospitalario de Toledo, 45071 Toledo, Spain; arodrigueza@sescam.jccm.es; 4 Department of Hematology, Complejo Asistencial Universitario de Salamanca (CAUSA), Instituto de Investigación Biomédica de Salamanca (IBSAL), Universidad de Salamanca (USAL), 37007 Salamanca, Spain; ana.marin.94@gmail.com (A.M.-Q.); jrgp@usal.es (J.R.G.-P.); jmbastida@saludcastillayleon.es (J.M.B.); 5 Grupo Español de Alteraciones Plaquetarias Congénitas (GEAPC), Sociedad Española de Trombosis y Hemostasia (SETH), 28006 Madrid, Spain

**Keywords:** congenital platelet disorders, inherited thrombocytopenias, platelet function disorders

## Abstract

Platelets play a major role in hemostasis as ppwell as in many other physiological and pathological processes. Accordingly, production of about 10^11^ platelet per day as well as appropriate survival and functions are life essential events. Inherited platelet disorders (IPDs), affecting either platelet count or platelet functions, comprise a heterogenous group of about sixty rare diseases caused by molecular anomalies in many culprit genes. Their clinical relevance is highly variable according to the specific disease and even within the same type, ranging from almost negligible to life-threatening. Mucocutaneous bleeding diathesis (epistaxis, gum bleeding, purpura, menorrhagia), but also multisystemic disorders and/or malignancy comprise the clinical spectrum of IPDs. The early and accurate diagnosis of IPDs and a close patient medical follow-up is of great importance. A genotype–phenotype relationship in many IPDs makes a molecular diagnosis especially relevant to proper clinical management. Genetic diagnosis of IPDs has been greatly facilitated by the introduction of high throughput sequencing (HTS) techniques into mainstream investigation practice in these diseases. However, there are still unsolved ethical concerns on general genetic investigations. Patients should be informed and comprehend the potential implications of their genetic analysis. Unlike the progress in diagnosis, there have been no major advances in the clinical management of IPDs. Educational and preventive measures, few hemostatic drugs, platelet transfusions, thrombopoietin receptor agonists, and in life-threatening IPDs, allogeneic hematopoietic stem cell transplantation are therapeutic possibilities. Gene therapy may be a future option. Regular follow-up by a specialized hematology service with multidisciplinary support especially for syndromic IPDs is mandatory.

## 1. Introduction

Inherited platelet disorders (IPD) comprise a heterogenous group of rare diseases caused by molecular anomalies in genes that are relevant in platelet formation and/or function. The relevance of clinical complications in patients with these diseases is highly variable, even within the same type, ranging from almost negligible to life-threatening. Consequently, the early and accurate diagnosis of patients and their close medical follow-up is long known to be of great importance [1]. At present, around 60 types of IPD due to molecular defects in about 75 different genes have been recognised [2]. The true prevalence of each is unknown and, while it is estimated that they affect between 1:10^4^ and 1:10^6^ individual, more moderate disorders are certainly more common, since oftentimes patients go unnoticed for many years and even their entire lives. Noteworthy, a recent survey of the frequency in the general population of molecular variants in genes associated with platelet disorders, has revealed that about 3 in 1000 subjects have a clinically meaningful loss-of-function variant in genes involved in IPDs [3].

**Figure 1:**
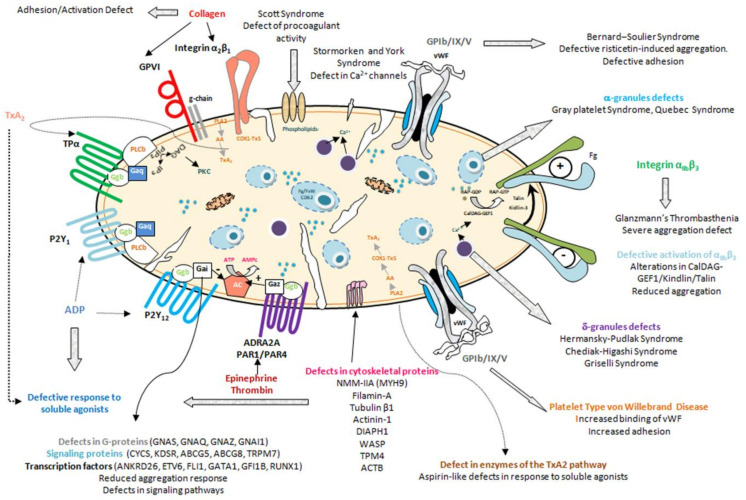
Inherited Platelet Disorders. The image shows the myriad of IPDs according to the protein and/or platelet function element which is affected by the genetic anomaly.

The two main groups of IPD are:

i. Inherited thrombocytopenias, in which the most conspicuous defect is the low number of circulating normal-sized, large, or small platelets [4,5,6] (Figure 1, Table 1)
ii. Inherited platelet function disorders, characterized by dysfunctional, typically hypofunctional, platelets resulting from defects of the membrane receptors, granules, elements involved in signal transduction, or other defects of the biochemical platelet machinery [7,8] (Figure 1, Table 2, Table 3 and Table 4)

Thrombocytopenia and thrombocytopathy are typically associated.

The common feature of IPD is a predisposition toward spontaneous mucocutaneous bleeding (epistaxis, gum bleeding, purpura, menorrhagia) beginning in childhood, which is usually moderate although it can worsen in situations of hemostatic compromise, such as trauma, drug treatments, surgeries, or childbirth. Less frequently, deep, severe bleeding can also occur (central nervous system hemorrhage, digestive bleeding) [1,119,120]. Likewise, many of these IPD comprise part of multisystemic disorders known as syndromic IPD. In some, bleeding may be clinically irrelevant, but patients display or are at high risk of presenting relevant disorders of other organs or tissues, or even neoplasms [1,5,7,121,122] (Table 1, Table 3 and Table 4). A relationship between genotype (the gene and type of molecular defect) and prognosis and/or clinical severity has been established in some IPD, making a molecular diagnosis especially imperative to guide clinical management [2,123]. Genetic diagnosis of IPD has been greatly facilitated by the introduction of high throughput sequencing (HTS) techniques into mainstream investigation practice in these diseases [124,125,126]. However, there still are unsolved ethical concerns on general genetic investigations as variants of unknown significance (VUS) and unexpected genetic defects can be found. Thus, patients should be informed in advance and comprehend the potential implications of such genetic findings [127,128].

In this review we will update the general approach to diagnosis of IPD, comment on some IPD that we deem most important given their relative frequency and/or clinical repercussions, and, finally, we will briefly mention management of and treatment options for these diseases. 

## 2. Inherited Platelet Disorder Diagnosis

The diagnosis of IPD has traditionally been limited by their considerable clinical and laboratory heterogeneity, as well as by the scant reproducibility and specificity of platelet function tests [129,130]. This has led to many patients reaching adulthood undiagnosed or misdiagnosed, thereby exposing themselves to a high risk of suboptimal clinical management or even harmful and unnecessarily invasive treatment. One clear example of this is the misdiagnosis of immune thrombocytopenia (ITP) in many individuals with Bernard-Soulier syndrome (BSS) and improper treatment with steroids or even splenectomy [119].

**Figure 2:**
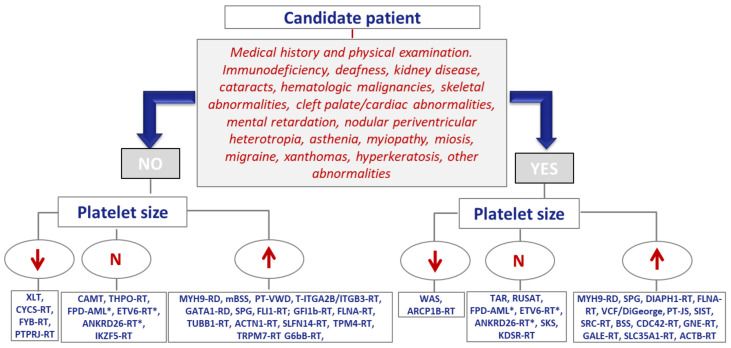
Diagnostic approach for inherited thrombocytopenias. N: Normal; XLT: X-Linked thrombocytopenia; CYCS-RT: CYCS-related thrombocytopenia (thrombocytopenia type 4); FYB-RT: FYB-related thrombocytopenia (adap-related thrombocytopenia); PTPRJ-RT: Thrombocytopenia due to variants affecting the phosphatase CD148; CAMT: Congenital amegakaryocytic thrombocytopenia (associated to a severe bone marrow aplasia ); THPO-RT: thrombocytopenia due to mutations in *THPO* encoding thrombopoietin; FPD-AML: Familial platelet disorder with propensity to acute myelogenous leukemia; ANKRD26-RT: Thrombocytopenia due to variants affecting ankyrin repeat domain 26; ETV6-RT: Thrombocytopenia due to variants in the transcription factor ETV6; IKZF5-RT: Thrombocytopenia due to variants in the transcription factor IKZF5; MYH9-RD: Disorders related to variants in MYH9 (neutrophil inclusions, deafness, kidney disease, cataracts); mSBS: monoalellic Bernard-Soulier syndrome (Mediterranean macrothrombocytopenia); PT-VWD: Platelet type von Willebrand disease; ITGA2B/ITGB3-RT: Thrombocytopenia due to variants in *ITGA2B/ITGB3*; GATA1-RD:GATA1–related disorders (commonly associated to red cells anomalies); GPS: Gray platelet syndrome (myelofibrosis); FLI1-RT: Thrombocytopenia due to variants in the transcription factor FLI1; GFI1-RT: Thrombocytopenia due to variants in *GFI1B* (abnormal CD34 expression in platelets); FLNA-RT: Thrombocytopenia with filaminopathy (syndromic or only thrombocytopenia); TUBB1-RT; Thrombocytopenia due to variants affecting tubulin b1; ACTN1-RT: Thrombocytopenia due to variants affecting Actinin-1; SLFN14-RT: SLFN14-Related thrombocytopenia; TPM4-RT: Thrombocytopenia due to variants in tropomyosin 4; TRPM7-RT: Thrombocytopenia related with the ionic channel TRPM7; G6b-B: Thrombocytopenia due to variants affecting the immunoreceptor G6b-B (myelofibrosis); WAS: Wiskott-Aldrich syndrome (eczema and severe infections; ARCP1B-RT: Mycrothrombocytopenia linked to a ARCPB; TAR: Thrombocytopenia with absent radii; RUSAT: Radioulnar synostosis with amegakaryocytic thrombocytopenia; SKS: Stormorken syndrome; KDSR-RT: Thrombocytopenia with variable hyperkeratosis due to variants affecting 3-dehydrosphinganine reductase; DIAPH1-RT: Thrombocytopenia associated to dominant deafness and variants in DIAPH1; PT-JS: Paris-Trousseau/Jacobsen syndrome; VCF-DiGeorge syndromes: velocardiofacial/DiGeorge syndrome; SIST: sistosterolemia (elevated plasma levels of plant sterols); SRC: Thrombocytopenia associated to gain-of-function variant in tyrosine kinase SRC; BSS: Bernard-Soulier syndrome; CDC42-RT: CDC42-related thrombocytopenia (Takenouchi-Kosaki syndrome); GNE-RT: GNE-related thrombocytopenia; GALE-RT: GALE-related thrombocytopenia (galactosemia); SLC35A1-RT: Syndromic thrombocytopenia due to variants affecting the CMP-sialic acid transporter; ACTB-RT: Thrombocytopenia due to variants affecting actin beta; * Disorders with high risk of hematological malignancy.

**Figure 3:**
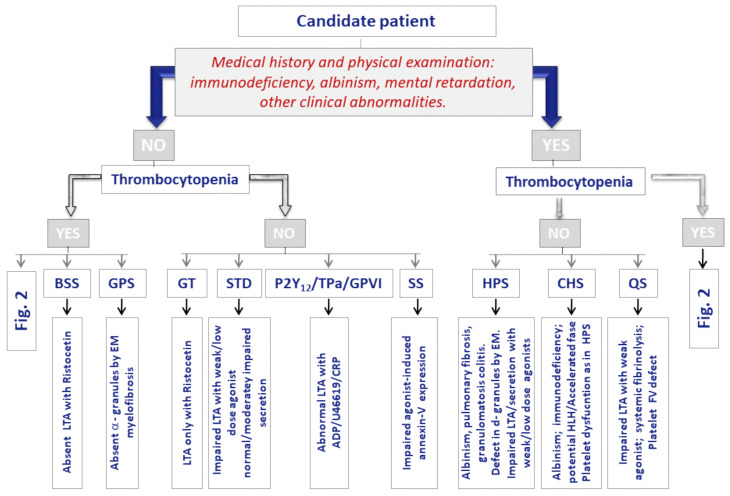
Diagnostic approach for suspected cases of inherited platelet function disorders. BSS: Bernard-Soulier Syndrome; GPS: Gray Platelet Syndrome; GT: Glanzmann thrombasthenia; STD: Signaling transmission defect (deficiencies in enzymes, G-proteins, CalDAG-GEF1, other signaling proteins); Specific defects in platelet receptors P2Y12 (ADP), Tpa (TxA2) or glycoprotein VI (GPVI) (collagen); SS: Scott Syndrome; HPS: Hermansky-Pudlak Syndrome; CHS: Chediak-Higashi Syndrome; QS: Quebec Syndrome. EM: electron microscopy; LTA: light transmission aggregometry; U46619:TxA2 analog; CRP: collagen-related peptide; Figure 2: Figure 2.

According to expert recommendations, the first-line diagnostic evaluation of IPD calls for: (i) comprehensive clinical investigation (including a physical examination paying special attention to signs of bleeding, the presence of extra-hemorrhagic syndromic alterations, and family history); (ii) laboratory analyses with general biochemical and coagulation testing, and (iii) complete blood count (CBC) and blood smear, focusing especially on platelet numbers and morphology. Analysis of the functional phenotype of the platelets, begin with the use of relatively basic, widespread, and largely nonspecific tests. Later, more definitive, complex methods are used that are generally only available in laboratories that are specialized in IPDs diagnosis [2,131]. Figure 2 and Figure 3 outline the diagnostic approach for inherited thrombocytopenias and inherited platelet function disorders, respectively, on the basis of the presence or absence of extra-hematological alterations and platelet size and function. Table 5 illustrates the stratification of the laboratory tests to study these IPD. 

Standardized scales or questionnaires, such as the International Society of Thrombosis and Haemostasis’ Bleeding Assessment Tool (ISTH-BAT) are recommended. The ISTH-BAT is a relatively straightforward tool that can be completed in 5 min [144]. Its usefulness in distinguishing IPD from von Willebrand disease (VWD) and from healthy controls has recently been assessed in a large cohort of 1098 subjects, 482 of whom were affected with an IPD (286 inherited thrombocytopenias and 196 inherited platelet dysfunction). The ISTH-BAT appears to be helpful when defining which patients require further functional studies to identify their disorder [120]. Broadly put, a ISTH-BAT >3 points in children, 4 in men, and 6 in women, is established to select cases with abnormal hemorrhagic diathesis who would be eligible for a more in-depth platelet function work-up for suspicion of thrombocytopathy. The ISTH-BAT was inaccurate in discriminating inherited thrombocytopenias from controls [120].

Many inherited thrombocytopenias display a moderately low platelet count and platelet dysfunction; clinical bleeding is very mild or absent. It is therefore not uncommon for thrombocytopenia to be an incidental finding in adulthood. Moreover, its genetic origin may not be suspected if there is no family history, either because it is recessively-inherited, or because, as documented in up to 40% of individuals with MYH9-RD (MYH9-related disease) it is due to molecular defects with incomplete penetrance or that appear *“de novo”* in the patient Fundamentals that inform the diagnosis of inherited thrombocytopenias include platelet size evaluation on the peripheral blood smear and a proper physical examination that points toward a syndrome. Platelet size (small, normal, large) tends to be characteristic of the different types (Figure 2) (Table 1), but it is worth remembering that exceptions do exist [145]. All of this increases the risk that ITP can be misdiagnosed, thereby exposing the patient to the risk of unnecessary treatments for years. Consequently, just as ITP is considered a diagnosis of exclusion, so too should inherited thrombocytopenias. 

The definitive diagnosis of IPD is reached by identifying the underlying molecular pathology. Until a decade ago, molecular study was deemed the last, non-essential step in the process of diagnosing IPD [130] and was performed almost solely by Sanger sequencing of the candidate genes identified according to patients’ clinical and laboratory phenotype [2,131]. This strategy, while being useful, is tedious and not applicable to cases with a non-specific phenotype [119], which is why it is being relegated to confirming molecular defects and family studies. After 2010, the molecular study of IPD begun to be undertaken by means of high throughput sequencing (HTS) of pre-selected gene panels (between 10 and 300 genes) or even the entire exome or genome. In just a few years, the use of this powerful technology has substantially broadened our knowledge of IPD, and HTS has identified the molecular base in almost two thirds of the approximately 60 known types of IPD [124,125,126,143,146,147,148,149]. In fact, the growing availability of this methodology at lower cost is changing the traditional IPD diagnostic process and many experts propose that molecular study be incorporated much earlier, even after the most basic initial testing [2,149]. 

Nowadays, early, accurate molecular diagnosis of IPD undoubtedly facilitates clinical management, particularly in the serious, potentially fatal types, in which genotype is related to prognosis and severity of hematological and/or extra-hematological disease, such as congenital amegakaryocytic thrombocytopenia (CAMT), familial platelet disorder with predisposition to hematological malignancies (FPD/AML), MYH9-RD, Wiskott-Aldrich syndrome (WAS), Hermansky-Pudlak syndrome (HPS) or Chediak-Higashi Syndrome (CHS) [8,26]. Nevertheless, HTS also has its limitations, such as managing the vast amount of molecular data obtained, which can only be addressed with the help of bioinformatics experts, and the accurate interpretation of the pathogenicity of the candidate variants [2,146,149]. 

Recognizing the potential value of identifying molecular defects, it must be remembered that incorrectly assigning molecular pathogenicity can also undermine clinical management. Therefore, expert recommendations must be followed when filtering and interpreting the pathogenicity of variants identified by HTS. Currently, the most widely used guideline is the American College of Medical Genetics and Genomics [150]. Likewise, despite the greater availability of HTS, the importance of the clinical evaluation and detailed analysis of the patients’ platelet phenotype cannot be ignored. Ethical issues also surround the use of HTS and shouldn’t be overlooked, such as properly informing patient before their molecular study or managing incidental molecular findings [127,128,151]. 

Given that IPD are rare diseases, collaboration between clinicians and researchers, both nationally and internationally, is indispensable to facilitate accessibility to early, accurate IPD diagnosis, enhance the definition of clinical phenotypes, establish clear-cut pathogenicity of new molecular variants, and to comprehend genotype-phenotype relationships better [146,149]. Accordingly, for more than a decade, we have been coordinating the multicenter project “Functional and molecular characterization of IPD patients” of the Spanish Group of Inherited Platelet Alterations (GEAPC, for its acronym in Spanish) of the Spanish Society of Thrombosis and Hemostasia (SETH). Its primary aim is to make accessible the specialized functional and molecular diagnosis of patients with IPD to all Spanish hematologists and, potentially, researchers from other countries [119,124]. Within the framework of this project, to date, close to 300 patients from some 200 unrelated families have been studied and about 60% of the cases have achieved molecular diagnosis. This is the largest case series collected in Spain and one of the largest worldwide. Additionally, the first Spanish registry of IPD has been launched (https://retplac.imib.es/ (accessed on 26 April 2021))

## 3. Inherited Platelet Disorders of Particular Clinical Relevance

Inherited platelet disorders with special clinical importance are discussed below, such as syndromic disorders caused by congenital defects and platelet disorders with a high degree of bleeding.

### 3.1. Syndromic Platelet Diseases

Approximately half of all inherited thrombocytopenias, as well as some inherited platelet function disorders, are syndromes in which the congenital platelet defect is associated with a high probability of clinically relevant alterations in other cell types, organs, or tissues, or with developing neoplastic disease [5,8,26,129]. In light of their considerable clinical relevance, we will highlight the following: 

#### 3.1.1. Congenital Amegakaryocytic Thrombocytopenia (CAMT)

This is a rare bone marrow failure that presents at birth as hypomegakaryocytic thrombocytopenia without any other physical characteristics. Most affected individuals develop additional cytopenias during childhood until finally progressing to bone marrow aplasia. 

This autosomal recessive disorder is caused by mutations in the *c-MPL* gene that provoke the expression of a dysfunctional thrombopoietin (TPO) receptor. There is a correlation between genotype and phenotype, such that the nonsense mutations display a more aggressive pattern with progression to hypocellular bone marrow within the first decade of life, whereas patients with amino acid substitution mutations can exhibit moderate decrease in platelet counts and later go on to develop anemia or pancytopenia. Allogeneic hematopoietic stem cell transplantation (allo-HSCT) is the only curative option for these patients [9].

A few cases (five unrelated families) have recently been identified with very similar clinical symptoms caused by biallelic mutations on the *THPO* gene that encodes TPO, leading to absent and/or dysfunctional circulating TPO [13]. Unlike patients with *c-MPL* mutations, individuals with molecular pathology in *THPO* fail to respond to allo-HSCT, given that the defect is extrinsic to the hematopoietic cells. In contrast, in these cases, administering thrombopoietic agents such as romiplostim, a TPO receptor agonist (TPO-RA) does induce trilinear hematopoietic responses, remission of bleeding and infections, and transfusion independence. On the other hand, the monoallelic mutations that result in loss of function, determine decreased circulating TPO and mild thrombocytopenia with autosomal dominant inheritance [12].

#### 3.1.2. MYH9-Related Disease (MYH9-RD)

These diseases are the leading cause of inherited thrombocytopenia worldwide, with more than 300 families identified. This category includes anomalies previously known as May-Hegglin, Fechtner, Sebastian, and Epstein syndromes. This autosomal dominant syndrome is caused by monoallelic mutations (some 100 different mutations) in the *MYH9* gene, which encodes for the non-muscle myosin heavy chain IIA chain (NMM-IIA protein) involved in platelet cytoskeletal contractility [55,56,152]. Noteworthy, *de novo* mutations are common in this disease and somatic germinal mosaicism has been reported [153].

Patients with MYH9-RD exhibit varying degrees of thrombocytopenia from birth; exceptional cases display normal numbers, but all clearly exhibit giant platelets. Often, albeit not always, neutrophilic inclusions similar to Döhle bodies are visible on the blood smear. These inclusions are more evident on immunofluorescence staining for NMM-IIA aggregates, a test currently used to diagnose this disease in specialized laboratories [152]. 

Although bleeding episodes tend to be rare and mild, and even absent, this is not a trivial disorder because 25% of the patients develop nephropathy with proteinuria that, in most cases, evolves into end-stage kidney failure and require dialysis or a kidney transplant. Furthermore, almost 50% of the patients will suffer neurosensorial deafness and 18%, presenile cataracts. Therefore, the clinical course of MYH9-RD is quite heterogeneous and ranges from asymptomatic, isolated thrombocytopenia to a complex disorder that severely affects quality of life. Should the need for preoperative increase in platelets arise, TPO-RA can be an acceptable option [154]. 

Research with large patient populations has identified genotype-phenotype correlations that aid in predicting how the disease will evolve in approximately 85% of the cases and, possibly strategies or treatments to prevent or delay kidney disease can be adopted. Few residues, as S96, R702, R1165 or D1424, are mutational hotspots. Generally, mutations affecting the N-terminal head domain of the protein are associated with a worse prognosis than those located on the C-terminal tail domain. By contrast, different mutations within the same domain, even affecting the same residue, such as D1424, can associate a significantly different risk of extra-hematologic manifestations that entail a very different prognosis [55,56]. 

#### 3.1.3. Wiskott-Aldrich Syndrome (WAS)

WAS is an X-linked disease that affects males almost exclusively, with clinical manifestations that include bleeding, eczema, and combined immunodeficiency (many patients have infections caused by opportunistic germs). Autoimmune syndromes have been observed in some 40% of the cases and patients have and increased risk of developing tumors, especially lymphoma, at any age. With the exception of extremely rare instances, one clinical hallmark of WAS is microthrombocytopenia, with counts of between 5 and 50 ×10^9^/L and small dysfunctional platelet (mean platelet volume [MPV]: 3.5-7 fL; normal: 7-11 fL) [83].

WAS is attributable to a molecular defect on the *WAS* gene (Xp11.22) that encodes the WASP protein that is involved in actin cytoskeletal remodeling. Four hundred different mutations have been reported in WAS patients, one third of which are located in nine gene hotspots. A relationship between clinical course and genotype has been reported, such that the mutations having the greatest effect on WASP expression/ functionality are associated with a more severe phenotype [83]. Hypomorphic mutations of the WAS gene can lead to an attenuated form of WAS called chronic or intermittent X-linked thrombocytopenia (XLT), which is correlated with a lower risk of the infections and malignancy, which otherwise are the leading cause of early death in the classical form of WAS.

The only curative treatment to date is allo-HSCT, with an 80% survival rate. Gene therapy comprises a promising approach for patients without a suitable donor. Immunoglobulin replacement therapy and oral antibiotics prevent infections and TPO-RA can be used to temporarily boost platelet counts in cases of severe refractory thrombocytopenia [83,84].

#### 3.1.4. Sitosterolemia (STSL)

Sitosterolemia, also known as phytosterolemia, is a rare autosomal recessive sterol storage disorder characterized by increased plant sterol levels in plasma. This disease results from recessive pathogenic variants in the *ABCG5* and *ABCG8* genes. The clinical characteristics of STSL include cutaneous and tendon xanthomas, xanthelasmas, premature coronary atherosclerosis and associated complications, arthritis, and/or arthralgias. 

Hematologic abnormalities, such as hemolytic anemia (with a negative direct antiglobulin test and stomatocytosis) and macrothrombocytopenia are present in 80-90% of the cases, and/or splenomegaly may also be present with other clinical attributes. Ezetimib (EZE) improves the distribution of VLDL and HDL, thereby lowering the atherogenic lipid profile, yielding a potential clinical benefit in STSL and increasing platelet count. A delay in diagnosis is not without consequences, as it can lead to inadequate clinical management with its incumbent risk of advanced atherosclerotic cardiovascular disease [57,58].

#### 3.1.5. DIAPH1-Related Thrombocytopenia (DIAPH1-RT)

DIAPH1-related thrombocytopenia is an autosomal dominant defect with macrothrombocytopenia and variable neutropenia, as well as hearing loss beginning in the first decade of life (at much younger ages than in MYH9-RD). DIAPH1, encoded by the homonymous gene *DIAPH1*, is a formin involved in the organization of the cytoskeleton of megakaryocytes and platelets, also present in the organ of Corti. 

In a recent multicenter study we have reported a 16-case series that reveals that patients exhibit megakaryocyte clustering in bone marrow, with deficient proplatelet formation. In in vitro cultures of megakaryocytes, these defects can be overcome, at least partially, with eltrombopag. This drug can enable DIAPH1-related thrombocytopenia to be temporarily corrected prior to surgery, thereby obviating platelet transfusion [66].

### 3.2. Inherited Platelet Disorders with Predisposition to Hematological Neoplasms

The WHO’s 2016 revision of the classification of myeloid neoplasms and acute leukemias [155] introduced a new category of diseases defined as myeloid neoplasms with germline predisposition and pre-existing platelet disorders, that includes those that evolve with molecular variants on ankyrin repeat domain 26 gene (*ANKRD26*) or on transcription factors *ETV6* and *RUNX1* [18,19,20,22,122,156,157].

These three autosomal dominant inherited disorders justify 18%, 3%, and 5%, of the inherited thrombocytopenias, respectively, and, together, account for almost one quarter of the cases. Individuals affected by these diseases typically present with mild, isolated, non-syndromic thrombocytopenia with normal-sized platelets. In all three conditions, bone marrow examination reveals a normal or increased number of megakaryocytes, typically with dysplastic characteristics, such as small hypolobulated nuclei. In patients with *ANKRD26*-RT (thrombocytopenia type-2) or *ETV6*-related disease (thrombocytopenia type-5), bleeding, when present, is generally mild, consistent with normal platelet function. In contrast, individuals with congenital *RUNX1*-related thrombocytopenia, known as familial platelet disorder with propensity to acute myelogenous leukemia (FPD/AML) typically exhibit platelet function abnormalities, primarily alpha/dense granules deficiency, that can associate a propensity toward bleeding of varying severity.

The common, clinically relevant characteristic of these three conditions is the greater risk of hematological neoplasms. Myelodysplastic syndrome (MDS) and acute myeloblastic leukemia (AML) develop in approximately 48% of patients with FPD/AML and in 7% of cases of *ANKRD26*-RT; 21% of individuals with *ETV6*-RT will go on to develop acute lymphoblastic leukemia (ALL), whereas another 7% will suffer from AML, MDS, multiple myeloma, or polycythemia vera. These malignacies generally debut between the second and fifth decade of life, while thrombocytopenia is generally present at birth. Only rarely will platelet counts be within the lower limit of normal range. Therefore, conspicuous thrombocytopenia is not an essential diagnostic criterion for these conditions, particularly in FPD/AML. 

### 3.3. Syndromic Disorders Due to Congenital Defects of Platelet Granules

Several congenital defects cause a quantitative or qualitative deficiency of platelet granules α, δ, or both (Table 3). Although patients with these conditions usually display a tendency toward moderate bleeding, some develop other highly relevant clinical complications [1,50,99]. 

Gray platelet syndrome (GPS) is a serious congenital deficit of α granules (number and/or content) ordinarily due to mutations in the *NBEAL2* gene [51,52]. People suffering from this autosomal recessive disorder usually present moderate thrombocytopenia, with large, pale platelets and moderate mucocutaneous hemorrhagic diathesis. This is a progressive syndrome, such that usually evolves towards severe thrombocytopenia during adolescence and adulthood, accompanied by a gradual degree of myelofibrosis and, in some cases, splenomegaly. Very recently, these patients have been reported to exhibit leukopenia, predisposition to autoimmune diseases, defective NETosis and to developing autoantibodies [51,53,158,159].

In addition to *NBEAL2*, other genes (*GATA1*, *VPS33B*, *VIPAS39, GFI1B, PLAU*) have been implicated in congenital α-granule deficiency [26]. Phenotypic differences among patients with mutations in these genes pose the controversy of whether hereditary disorders of α granule biogenesis should be characterized as GPS [160].

α-granule deficiencies may appear as a single phenomenon of unknown genetic cause or as one component of multisystemic diseases that affect all lysosome-like organelles. Among these deficiencies are Hermansky-Pudlak (HPS), Chediak-Higashi (CHS), and Griscelli (GS) syndromes, in which platelet numbers are typically normal and platelet function is moderately impaired. Consequently, moderate hemorrhagic diathesis is also common. Nevertheless, individuals with these diseases can present other complications that are clinically highly relevant [99]. 

HPS is a serious deficiency of dense granules, characterized by oculocutaneous albinism, accumulation of ceroid material in cells of the mononuclear phagocytic system, variable pulmonary fibrosis, inflammatory intestinal disease, and hemorrhagic diathesis. Its molecular basis is heterogenous, with mutations in up to 11 different genes (Table 3), all of which participate directly or indirectly in intracellular vesicular traffic. A relation between the affected gene and clinical severity has been proven (particularly in HPS subtypes 1 and 4), making early molecular diagnosis particularly important [102,103,104]. 

CHS is caused by mutations in the *LYST* gene, which encodes the lysosomal trafficking regulating protein CHS1 (or LYST). It manifests with partial oculocutaneous albinism, silver hair, giant lysosomal granules, inclusion bodies in neutrophils and other cells, frequent pyogenic infections, peripheral neuropathy, and accelerated phase in up to 85% of the cases. Allo-HSCT is the only curative option in severe cases [106]. 

GS presents with partial albinism, silver hair, central neurological defects, and/or lymphohistiocytosis. It arises from mutations in genes encoding the proteins MYO5A, RAB27A, or melanophilin, involved in organelle traffic. Platelet counts are usually normal, but platelet function is moderately altered [50,99]. 

### 3.4. Platelet Disorders with High Risk of Severe Bleeding

In a small number of IPD, there is severe platelet dysfunction, sometimes combined with thrombocytopenia, which conditions a high risk of clinically serious, even life-threatening, bleeding, particularly in situations of high hemostatic compromise (trauma, surgeries, childbirth). We will focus here on the most widely characterized disorders, Bernard-Soulier syndrome (BSS) and Glanzmann thrombasthenia (GT).

#### 3.4.1. Defects of the GPIb/IX/V Complex

BSS is an autosomal recessive hemorrhagic diathesis characterized by moderate or severe thrombocytopenia and giant, dysfunctional platelets. It is due to mutations (more than 100 different mutations reported) in *GP1BA*, *GP1BB*, and *GP9* genes, leading to the absence of the Ib/IX/V complex in platelets (classical BSS) or, in exceptional cases, the expression of a non-functional receptor (variant BSS) [31]. The most unique laboratory anomaly of BSS is the absence of platelet agglutination with ristocetin, which is not corrected with normal plasma, unlike severe von Willebrand disease (VWD). In contrast, aggregation with other agonists (adenosine diphosphate [ADP], collagen, adrenaline, thrombin, etc.) is normal. Flow cytometry enables rapid detection of the selective deficit of the Ib/IX/V receptor and increased MPV. 

BSS must be, early in life, differentiated from other macrothrombocytopenias and from ITP to avoid inappropriate therapies or splenectomy [119]. Ordinarily, only homozygous patients display relevant mucocutaneous bleeding beginning in childhood, whereas heterozygous individuals (monoallelic BSS) tend to be asymptomatic with mild thrombocytopenia [1,31,35].

Platelet-type von Willebrand disease (PT-VWD), is also caused by rare mutations (only five reported) in the *GP1BA* gene. The effect of these autosomal dominant mutations is an extremely high affinity of the Ib/IX/V receptor for the VWF. This translates as platelet hyperresponsiveness to the antibiotic ristocetin, and as a low ratio between functional activity and plasma VWF antigen level (VWF:RCo/VWF:Ag < 0.6), reflecting the loss of high molecular weight multimers of VWF. The differential diagnosis of PT-VWD and VWD type IIB is very important, given that both have a similar clinical and laboratory phenotype, but patients require very different treatments [36].

A heterozygous deletion of chromosome 22q11.2, mostly somatic, including *GP1BB* causes Di-George and velocardiofacial syndromes in which macrothrombocytopenia is accompanied by several developmental defects [161]. 

#### 3.4.2. Glanzmann Thrombasthenia

In the hemostatic response, platelets adhered to the vascular wound recruit other circulating platelets to form the thrombus [162]. This platelet aggregation is achieved by means of fibrinogen bridges between αIIbβ3 integrins of nearby platelets. Molecular defects of the *ITGA2B* and *ITGB3* genes, that encode for the αIIbβ3 complex, cause GT. Some 200 different mutations (deletions, insertions, point mutations, etc.) have been reported in approximately 200 families, and these numbers are growing constantly [91,92,163].

Individuals with GT display normal platelet count and morphology, as well as strikingly defective aggregation to multiple agonists (ADP, collagen, arachidonic acid, and thrombin), whereas agglutination with ristocetin is normal or moderately decreased in its second wave. Flow cytometry reveals the deficit of the αIIbβ3 receptor. According to the platelet expression level of the integrin, there are three types of GT: i) Type I, with a total absence (<5%) of αIIbβ3; ii) Type II, with 10–20% of residual αIIbβ3; iii) variant GT, with non-functional expression (>50%) of αIIbβ3. 

Because it is a recessive autosomal disorder, only homozygote subjects exhibit moderate-severe mucocutaneous bleeding starting in childhood, whereas heterozygote individuals tend to be asymptomatic [1,8,163]. On the other hand, some *ITGA2B* and *ITGB3* heterozygous variants can cause autosomal dominant inherited thrombocytopenia [39].

The study of the incidence of cardiovascular and inflammatory diseases, cancers, and other conditions in patients with GT can contribute to our understanding of the role of integrin αIIbβ3 and αvβ3 in human health [90].

## 4. Management of Patients with Inherited Platelet Disorders

In the last few years, there have been no major changes in the clinical management of IPD patients and the recommendations issued years ago are still valid [1]. These include educational and preventive measures, first and foremost to minimize their risk of bleeding (avoiding activities with risk of trauma, good dental hygiene, no antiplatelet drugs), inclusion in registries, and regular follow-up by a specialized hematology service with multidisciplinary support especially for syndromic IPD.

Depending on their severity or level of risk, hemorrhagic complications and situations that entail bleeding risk are managed with hemostatic drugs, such as anti-fibrinolytics or desmopressin (DDAVP), and, when necessary with recombinant factor VII (rFVIIa) or with platelet transfusions.

rFVIIa acts by increasing thrombin generation. It is licensed and mainly used in GT, particularly in patients refractory to treatment with platelet concentrates [164,165,166,167]. There is scant evidence for the remaining platelet syndromes, but it has been successfully used to treat or prevent bleeding in few patients with BSS and a patient with thrombocytopenia-absent radii (TAR) [5,165,168,169]. 

Given the high risk of alloimmunization, platelet transfusion should be restricted to severe bleeds in which antifibrinolytics and local measures fail, or in patients with very severe thrombocytopenias who are to undergo major surgery or during childbirth. Leukodepleted single donor and/or HLA-identical platelet products are preferable for IPD patients. Overall, platelet transfusion should be indicated on a case-by-case basis, assessing risk-benefit and bearing in mind personal history and platelet count [1,164,165,170]. In a recent international study, surgical hemorrhage was shown to be common in patients with IPD (19.7%), with a significantly higher incidence of bleeding in individuals with inherited platelet disorders (24.8%) than in people with inherited thrombocytopenias (13.4%) [167]. It is paradoxical that, although the most commonly used prophylaxis was platelet transfusion, DDAVP, alone or with antifibrinolytics, was the preventive treatment associated with the lowest bleeding rates. Moreover, platelet transfusions in these patients were only effective when more than 6 units of platelets were transfused [167]. In this regard, recent studies suggest that in diseases with significantly dysfunctional platelets (such as GT), a critical proportion of approximately 2:1 of endogenous (dysfunctional) to transfused (healthy) platelets is essential to prevent prolonged bleeding induced by hemostatic lesions [171]. 

When deemed necessary to improve thrombocytopenia, such as in high bleeding risk surgery, treatment can involve platelet transfusion or, in some cases, TPO-RA (eltrombopag, romiplostin) [1,7,164,165,166,167,170,172,173] (reviewed in Bastida el al in this special issue). 

Insofar as inherited syndromic thrombocytopenias are concerned, a multidisciplinary clinical approach is essential. MYH9-RD patients may benefit from regular urine analysis, since appearance of proteinuria may support the use of therapies such as angiotensin II receptor blockers (ARB-II) and/or angiotensin-converting enzyme inhibitors (ACEI), aiming to prevent or delay kidney failure. MYH9-RD patients also benefit from routine hearing and eye examinations to avoid treatment delays; both cochlear implant and standard cataract surgery generally restore the function of both organs [55,121,174,175]. 

Insofar management for thrombocytopenias associated with *RUNX1*, *ANKRD26*, or *ETV6* mutations are concerned, a cytogenetic bone marrow examination is recommende at the time of diagnosis, particularly if the mutations have already been reported and have proven pathogenicity. If no significant finding are identified, annual CBC, blood smear, and clinical evaluation are suggested, so as to identify early signs of malignant disorders. In this case, if allo-HSCT is under consideration potential family stem cells donors must be tested and ruled out for the molecular alteration of the patient [18,20,22,121].

Though many IPD can be regarded as benign in the sense that they can be relatively well managed with support therapies, in some cases, bleeding or other complications are very severe, to the point of being life-threatening. As with other diseases, a definitive cure for IPD continues to be a challenge. To date, allo-HSCT is the only curative therapy clinically available for IPD cases with very severe bleeding symptoms, with high risk of transformation to bone marrow aplasia, or with hematological malignancy. Considering the high morbidity (especially graft-versus-host disease) and mortality, the risk-benefit analysis of performing allo-HSCT must be consideren on an individualized basis. In the event that it is contemplated, allo-HSCT is best carried out in childhood because of the lower risk of related complications [1,5,164,165,170,176]. 

Conditions in which HSCT therapy has been proven to be a main and highly effective option are classic CAMT and WAS [5,9,177,178,179]. HSCT has also been performed in sporadic cases of patients with MECOM-associated syndrome presenting and bone marrow failure [15], thrombocytopenia with radioulnar synostosis (RUSAT1), thrombocytopenia with absent radii (TAR), BSS, GT, GPS, and CHS, and could be appropriate in patients with FPD/AML or *ANKRD26*-RT or *ETV6*-RT who develop hematological neoplasms [5,18,22,106,166].

Gene correction is long expected to become a curative treatment for severe IPD. To date, it has been an experimental clinical option only for some cases of WAS, treated with autologous hematopoietic stem cells transduced with self-inactivating lentiviral vector encoding WAS protein. In these cases, all but one in pediatric age, sustained cell engraftment was achieved and platelet counts and function, bleeding and immunity improved significantly with no relevant vector-related toxicity [180,181,182,183]. For other IPD, however, gene therapy still is at pre-clinical research level [184]. Previous studies in cells, mice and dog models have shown the effectiveness of lentiviral vectors to drive the expression of the therapeutic transgene aimed to correct severe IPD such as GT and BSS [185,186,187,188]. The next step to take before clinical application is the demonstration that a designed lentiviral vector can correct the defective phenotype in human cells. Recently, we have generated GT-like CD34^+^ cells by disrupting the *ITGB3* gene in healthy CD34+ cells using two synthetic guide RNAs taking advantage of the CRISPR/Cas9 technology. Differentiation of these GT-like CD34^+^ cells towards the megakaryocytic lineage shows up to 90% decreased expression of both CD61 and CD41. Studies undergoing in this model show that transduction of GT-like CD34^+^ cell with a self-inactivating lentiviral vector in which the *ITGB3* gene is driven by the human glycoprotein 6 promoter (hGP6.*ITGB3* LV) re-expressed CD61 and CD41 in in vitro differentiated megakaryocytes (unpublished data). These good results suggest that this vector may enable the phenotypic correction of GT megakaryocytes, and re-open the potential applicability of gene therapy in GT patients. 

Interestingly, a recent study has provided in vitro proof-of-principle that CAMT patients can be treated by gene editing modern methods to correct the pathogenic MPL mutations in patient-derived hematopoietic stem cells [189].

## 5. Conclusions

To date, around 60 types of IPD due to molecular disease in about 75 different genes are known. The true prevalence of each type is unknown, but the overall frequency in the general population can reach up to 3 in 1000 subjects. While some IPD may remain asymptomatic for years, even during the lifetime, complications can arise upon hemostatic challenges. In others cases, IPD present relevant bleeding complications since birth, are syndromic diseases or predispose to malignancy. Therefore, early and accurate diagnosis, including genetics, and close medical, follow-up, including multidisciplinary approaches in syndromic IPD, are of great importance for preventive and therapeutic managements of IPD patients. Further basic and clinical research, including development of safe and potentially curative treatments, will benefit IPD patient’ quality of life and life expectancy.

## Figures and Tables

**Table 1:** General features of inherited thrombocytopenias.

Disease (OMIM Code)	Inh.	Gene	Type Tcp/S/HM	Clinical & Laboratory Phenotype in Most Reported Cases	Ref.
**INHERITED THROMBOCYTOPENIAS WITH NORMAL PLATELET SIZE**					
Congenital amegakaryocytic thrombocytopenia (CAMT) (604498)	AR	*MPL*	S	Very severe neonatal thrombocytopenia, amegakaryocytic; progression to aplastic anemia in childhood. Severe bleeding tendency.	[9,10]
*THPO*-related thrombocytopenia	AD/AR	*THPO*	S	Mono-allelic mutations are associated with mild thrombocytopenia. Bialellic mutations resemble CAMT. It does not respond to allo-HSCTH but it responds to Romiplostim. Severe bleeding tendency.	[11,12,13]
Radioulnar synostosis with amegakaryocytic thrombocytopenia 1 (RUSAT1, 605432)	AD	*HOXA11*	S	Severe neonatal thrombocytopenia; reduced/absent megakaryocytes. Possible evolution to aplastic anemia in childhood. Radius and ulna synostosis with/without other skeletal alterations; probable sensorineural hearing loss. Severe bleeding tendency.	[5,14]
*MECOM*-related thrombocytopenia (including amegakaryocytic thrombocytopenia 2 with Radioulnar synostosis, (RUSAT2) (616738)	AD	*MECOM*	S	Severe neonatal thrombocytopenia. Reduced/absent megakaryocytes and/or hyporegenerative anemia. Radius and ulna synostosis with/without other skeletal alterations; B-cell deficiency. Possible renal or cardiac malformations and probable sensorineural hearing loss. Severe bleeding tendency.	[15,16]
Thrombocytopenia with absent radii (TAR) (274000)	AR	*RBM8A* (Microdeletion & rs139428292/ rs201779890)	S	Moderate-severe central neonatal thrombocytopenia that improves with age; bilateral absence of radius with or without other skeletal abnormalities. Potential kidney, cardiac or central nervous system anomalies. Severe bleeding tendency.	[17]
Familial platelet disorder with propensity to acute myelogenous leukemia (FPD-AML) (601399)	AD	*RUNX1*	HM	Mild-moderate neonatal thrombocytopenia. Platelet function defect “Aspirin-like”. Platelet granule deficiency. High risk (>40%) of acute myeloblastic leukemia or myelodysplastic syndrome at a young age; increases risk or lymphoblastic leukemia and solid tumor. Absent to moderate bleeding tendency.	[18,19]
*ANKRD26*-related thrombocytopenia (thrombocytopenia Type-2) (188000)	AD	*ANKRD26*	HM	Mild-moderate neonatal thrombocytopenia. Some patients with high levels of hemoglobin and/or leukocytosis. Approximately 10% of patients acquire myeloid neoplasms. Absent to mild bleeding tendency.	[18,20,21]
*ETV6*-related thrombocytopenia (thrombocytopenia Type-5) (616216)	AD	*ETV6*	HM	Red blood cells with high mean corpuscular volume. Platelets may show elongated α granules, impaired spreading and clot retraction. High number of circulating CD34 + cells. Predisposition (30%) to acquired lymphoid, myeloid, and myeloproliferative syndromes. Absent to mild bleeding tendency.	[18,22,23]
*IKZF5*-related thrombocytopenia	AD	*IKZF5*	Tcp	Mild to moderate thrombocytopenia. Platelets with fewer α and δ granules. No bleeding tendency.	[24]
*CYCS*-related thrombocytopenia (thrombocytopenia type 4) (612004)	AD	*CYCS*	Tcp	Mild to moderate thrombocytopenia. May also present with small platelets. No bleeding tendency.	[25,26]
*KDSR*-related thrombocytopenia	AR	*KDSR*	S	Early severe thrombocytopenia (although the platelet count may be normal at birth). Platelet secretion and activation defect. Variable hyperkeratosis from palmoplantar and anogenital hyperkeratosis/erythema to a Harlequin ichthyosis. Moderate to severe bleeding tendency.	[27,28]
Stormorken syndrome (185070)	AD	*STIM1* *ORAI1*	S	Moderate thrombocytopenia. High systemic platelet binding of annexin V. Abnormal clot formation and retraction; asplenia, mild anemia, myopathy with tubular aggregates, congenital miosis, ichthyosis, short stature, migraine and mild cognitive impairment. Mild bleeding tendency.	[26,29]
York platelet syndrome	AD	*STIM1*	S	More rare than Stormorken syndrome. Moderate to severe thrombocytopenia. Myopathy with rimmed vacuoles; Platelets with fewer α and δ granules; giant dense bodies. Mild bleeding tendency.	[30]
**INHERITED THROMBOCYTOPENIAS WITH LARGE PLATELETS**					
Bernard-Soulier syndrome (BSS) (231200]	AR	*GP1BA, GP1BB*, *GP9*	Tcp	Moderate to severe thrombocytopenia and giant platelets. Absence of platelet aggregation with ristocetin, not restored with plasma, due to absence of severe dysfunction of the main vWF platelet receptor Ibα/IX. Normal/reduced response to other agonists. Defect of adhesion to FVW. Severe bleeding tendency.	[31,32]
Bernard-Soulier syndrome, monoallelic form (mBSS) (153670) (previously referred to as Mediterranean thrombocytopenia)	AD	*GP1BA, GP1BB*	Tcp	Mild to moderate thrombocytopenia. Absent to mild bleeding tendency.	[33,34,35]
Platelet-type von Willebrand disease (PTvWD) (177820)	AD	*GP1BA*	Tcp	Mild to severe thrombocytopenia. Platelet count can be normal but decrease under stress conditions. Platelet aggregates in blood smear. Abnormally high platelet VWF binding and hyperaggregation with low-dose ristocetin due to gain-of–function variants in GPIbα. Aggregation of washed platelets inducible with cryoprecipitate (not in VWD type 2B). Absence of high molecular weight multimers (as in VWD type 2B). Absent to mild bleeding tendency.	[36]
*ITGA2B*/*ITGB3*-related thrombocytopenia (187800)	AD	*ITGA2B, ITGB3*	Tcp	Mild to moderate thrombocytopenia. Defective expression and αIIbβ3 activation. Moderate bleeding tendency. Some monoallelic variants in *ITGA2B/ ITGB3* mutations cause constitutive activation of integrin αIIbβ3 with a phenotype similar to variant Glanzmann’s thrombasthenia.	[37,38,39]
DiGeorge/velocardiofacial syndrome	AD	Deletions in 22q11.2 (*TBX1/GP1BB,* deletions)	Yes	Moderate thrombocytopenia. Heart abnormalities, parathyroid, and thymus insufficiency, cognitive delay, facial dysmorphia. Most patients have developmental differences. Mild to severe bleeding tendency. Bleeding diathesis can be contributed not only by platelet dysfunction (absent in some cases) but also by other alterations such as velopharyngeal insufficiency, chronic nasal irritation, nasal allergies, or abnormal vWF multimers.	[40]
Jacobsen syndrome (147791), Paris-Trousseau thrombocytopenia (188025)	AD	Deletions in 11q23	S	Moderate to severe thrombocytopenia; may improve over time. Cardiac, facial, urinary tract, kidney, or central nervous system malformations; mental retardation; giant platelet granules. Mild to severe bleeding tendency.	[41,42]
*FLI1*-related thrombocytopenia, (61744)	AD/AR	*FLI1*	Tcp	Mild to moderate thrombocytopenia. Large platelet granules. Absent to moderate bleeding tendency (monoallelic and biallelic forms, respectively).	[43,44]
*GATA1*-related disorders (314050; 300367)	XL	*GATA1*	S	Mild to severe thrombocytopenia. Can associate with dyserythropoiesis with or without anemia, β-thalassemia, neutropenia, splenomegaly or congenital erythropoietic porphyria); Dysplastic megakaryocytes. Platelets granule deficiency and functional defect. A very rare form associates with blood group Lu a- b- (Lu null). Mild to severe bleeding tendency.	[45,46]
*GFI1b*-related thrombocytopenia (187900)	AD/AR	*GFI1B*	Tcp	Mild to severe thrombocytopenia (monoallelic & biallelic forms). Red blood cells with anisopoikilocytosis, dysplastic megakaryocytes, emperipolesis. Platelets with α/β granule deficiency and aggregation defect. CD34+ abnormal expression in platelets. Absent to severe bleeding tendency (monoallelic & biallelic forms).	[47,48,49]
Gray platelet syndrome (GPS) (139090)	AR	*NBEAL2*	S	Moderate to severe thrombocytopenia with typically grayish platelets. Could aggravate over time. Selective absence of α-granules demonstrable by electron microscopy; defect of α-granular proteins by biochemical assays (β-TG, PDGF, vWF, etc.). Impaired platelet function with weak agonists. In some patients, selective defects of GPVI and activation by collagen. Elevated vitamin B12 serum level. Early myelofibrosis; occasional splenomegaly; predisposition to autoimmune diseases. Moderate to severe bleeding tendency.	[50,51,52,53]
G6b-B-related Thrombocytopenia (617441)	AR	*MPIG6B*	S	Mild to severe thrombocytopenia; atypical megakaryocytes. Microcytic anemia, leukocytosis, myelofibrosis. Mild to moderate bleeding tendency.	[54]
*MYH9*-related disease, (*MYH9*-RD) (155100)	AD	*MYH9*	S	Mild to severe thrombocytopenia; In some cases, basophilic neutrophilic inclusions (Döhle bodies), hearing loss, kidney disease, liver disease, cataracts; Strong genotype–phenotype relationship. Absent to mild bleeding tendency.	[10,41,42,55,56]
Thrombocytopenia associated with sitosterolemia	AR	*ABCG5*, *ABCG8*	S	Mild to moderate thrombocytopenia. Elevated plasma levels of plant sterols. Xanthomas in tendons and xanthelasmas. Premature atherosclerosis; hemolytic anemia with stomatocytosis. Also non-syndromic picture. Mild to moderate bleeding tendency.	[57,58]
*FLNA*-related thrombocytopenia	X-linked	*FLNA*	S	Moderate thrombocytopenia. Nodular periventricular heterotopia; skeletal malformations, mental retardation, heart valve dystrophy, intestinal obstruction, bone dysplasia. May associate with Ehlers-Danlos syndrome; May also be nonsyndromic thrombocytopenia. Mild bleeding tendency.	[59,60,61]
*TUBB1*-related thrombocytopenia (613112)	AD	*TUBB1*	Tcp	Mild to moderate thrombocytopenia. In some patients, normal platelet count with increased platelet size. Absent to mild bleeding tendency.	[26,62,63]
*ACTN1*-related thrombocytopenia (615193)	AD	*ACTN1*	Tcp	Thrombocytopenia and moderate platelet dysfunction. Mild to moderate thrombocytopenia. Some giant platelets can be seen. Absent to mild bleeding tendency.	[26,64,65]
DIAPH1-related thrombocytopenia	AD	*DIAPH1*	S	Mild thrombocytopenia (occasionally normal platelet counts); moderate fluctuating neutropenia with low to moderate infectious risk; early sensorineural deafness. Absent to mild bleeding tendency.	[66]
*SLFN14*-related thrombocytopenia (616913)	AD	*SLFN14*	Tcp	Mild to moderate thrombocytopenia. δ granule secretion defect. Increased number of immature platelets. Moderate to severe bleeding tendency.	[67,68]
*SRC*-related thrombocytopenia (*SRC*-RT) (thrombocytopenia type-6) (616937)	AD	*SRC*	S	Mild to severe thrombocytopenia. Platelet granule defect, myelofibrosis and early edentulism, facial dysmorphia. High number of megakaryocytes, with immature features such as hypolobulated nuclei. Mild to severe bleeding tendency.	[69,70,71]
*TPM4*-related thrombocytopenia	AD	*TPM4*	Tcp	Mild thrombocytopenia. Absent to mild bleeding tendency.	[72]
*TRPM7*-related thrombocytopenia (*TRPM7*-RT)	AD	*TRPM7*	Tcp	Moderate thrombocytopenia. Absent to mild bleeding tendency	[73]
*PRKACG*-related thrombocytopenia (*PRKACG*-RT) (616176)	AR	*PRKACG*	Tcp	Severe macrothrombocytopenia. PKA-dependent protein phosphorylation defect (filamin A, GPIbβ). Severe bleeding tendency.	[74]
Takenouchi-Kosaki syndrome with macrothrombocytopenia (616737)	AD	*CDC42*	S	Moderate thrombocytopenia. Defective intellectual, growth, and psychomotor development. Possible brain, facial/ muscle/skeletal abnormalities. Also, immunodeficiency, eczema, hearing/visual disability, lymphedema, and cardiac or genitourinary malformations. No bleeding tendency.	[75,76]
*GNE*-related thrombocytopenia	AR	*GNE*	S	Severe thrombocytopenia. Myopathy with rimmed vacuoles since early adulthood. Isolated thrombocytopenia some patients. Increased reticulated platelets, suggesting accelerated platelet clearance. Mild to severe bleeding tendency.	[77,78,79]
*GALE*-related thrombocytopenia	AR	*GALE*	S	Severe thrombocytopenia. Galactosemia, anemia and febrile neutropenia. Severe bleeding tendency.	[78,80]
*SLC35A1*- related thrombocytopenia	AR	*SLC35A1*	S	Moderate thrombocytopenia. Impaired psychomotor development, epilepsy, ataxia, microcephaly, and choreiform movements. Mild bleeding tendency.	[26,81]
*ACTB*- related thrombocytopenia	AD	*ACTB*	S	Mild to moderate thrombocytopenia. Leucocytosis with eosinophilia or leukopenia. Other possible features are microcephaly, minor facial anomalies, developmental delay, and mild intellectual disability. No bleeding tendency.	[82]
**INHERITED THROMBOCYTOPENIAS WITH SMALL PLATELETS**					
Wiskott-Aldrich syndrome (WAS)(301000)	XL	*WAS*	S	Severe thrombocytopenia. Immunodeficiency, eczema, lymphoproliferative, and autoimmune disorders. Severe bleeding tendency.	[83,84]
X-linked thrombocytopenia (XLT or thrombocytopenia type 1) (313900)	XL	*WAS*	S	Moderate to severe thrombocytopenia. Mild form of Wiskott-Aldrich. Possible immunodeficiency and mild eczema. Increased risk of malignancy and autoimmune disorders. Absent to moderate bleeding tendency.	[83,84]
*FYB*-related thrombocytopenia (ADAP-related thrombocytopenia or (thrombocytopenia type 3) (273900)	AR	*FYB*	TCP	Moderate to severe thrombocytopenia. Baseline platelet hyperactivation. Impaired activation of αIIbβ3. Mild to moderate bleeding tendency.	[85,86]
ARCP1B-related thrombocytopenia (617718)	AR	*ARCP1B*	S	Moderate to severe thrombocytopenia. Normal platelet count in some cases. Eosinophilia, immune-mediated inflammatory disease, eczema, lymphadenopathy, hepato-splenomegaly. Impaired growth. Moderate bleeding tendency.	[87,88]
PTPRJ-related thrombocytopenia (CD148-related thrombocytopenia) (*PTPRJ*-RT, na)	AR	*PTPRJ*	Tcp	Moderate to severe thrombocytopenia. So far, a single pedigree with PTPRJ-RT has been identified. Impaired platelet reactivity with GPVI agonists (collagen, CRP, convulxin). SRC-type kinase activation defect. Megakaryocyte maturation defect. Mild to moderate bleeding tendency.	[89]

Inh: inheritance; AD: autosomal dominant; AR: autosomal recessive; XL: chromosome X-linked; Tcp: IT with mainly isolated thrombocytopenia; S: IT associated with other syndromes or additional diseases; HM:IT predisposing to hematological malignancies and/or solid neoplasm; HSCT: Allogeneic hematopoietic stem cell transplantation.

**Table 2:** Inherited defects of platelet receptors.

Receptor	Disease (OMIM Code)	Inh.	Genes	Clinical & Laboratory Phenotype in Most Reported Cases	Ref.
Ib/IX/V	Bernard-Soulier Syndrome (SBS) (231200)	AR	*GP1BA*, *GP1BB*, *GP9*	See Table 1.	-
	Platelet Type von Willebrand Disease (PT-vWD) (177820)	AD	*GP1BA*	See Table 1.	-
α_IIb_β_3_	Glanzmann Thrombasthenia (GT) (273800)	AR	*ITGA2B*, *ITGB3*	Normal platelet count and morphology. Absent or severely reduced LTA with all agonists (ADP, TxA2, collagen, thrombin). LTA with ristocetin normal or 2nd wave reduced. Absent or severely reduced clot retraction. Absence or decreased αIIbβ3 expression demonstrable by flow cytometry: Type I <5%; Type II 10–20%; Variant GT with even >50% non-functional αIIbβ3. Severe bleeding tendency.	[26,90,91,92]
P2Y12	ADP receptor deficiency (609821)	AD/AR	*P2RY12*	Normal platelet count and morphology. LTA greatly decreased with ADP. With other agonists normal or reduced second wave. Absent ADP inhibition of PGE1-induced cAMP synthesis. Defective VASP de-phosphorylation with ADP. Mild to moderate bleeding tendency. Bleeding mostly associated with antiplatelet intake, dental interventions, or other hemostatic challenges.	[8,93,94]
TxA_2_ –R (TPα)	TxA_2_ receptor deficiency (614009)	AD/AR	*TBXA2R*	Normal platelet count and morphology. Absent or severely reduced LTA with arachidonic acid or TxA2 analogs such as U46619 or STA2. Normal with other agonists or reduced second wave. Mild to moderate bleeding tendency. Bleeding mostly associated with antiplatelet intake, dental interventions, or other hemostatic challenges. Post- surgery bleeding.	[8,95]
GPVI	GPVI collagen receptor defect (614201)	AR	*GP6*	Normal platelet count and morphology. Absent or severely reduced LTA with collagen and GPVI agonists such a as collagen related-peptide or convulxin. Also defective platelet secretion and protein tyrosine phosphorylation with GPVI agonists Mild to moderate bleeding tendency. Bleeding mostly associated with antiplatelet intake, dental interventions, or other hemostatic challenges.	[8,96,97]
EPHB2	Ephrin type-B receptor 2 defect (618262)	AR	*EPHB2*	Normal platelet count and morphology. Impaired LTA and secretion with multiple agonists. Impaired GPVI pathway signaling and inside-out αIIbβ3 signaling. Moderate to severe bleeding tendency.	[98]

Inh: inheritance; AD: autosomal dominant; AR: autosomal recessive; LTA: light transmission aggregometry; vVF: von Willebrand factor; GPs: flycoproteins; vVD: von Willebrand disease; ADP: Adenosin difosphosphate; P2Y12: ADP receptor P2Y12; TxA_2_: Tromboxane A_2._

**Table 3:** Inherited defects of platelet granules.

Granule	Disease (OMIM code)	Inh.	Genes	Clinical & Laboratory Phenotype in Most Reported Cases	Ref.
α and δ	Idiopathic granule deficiency	AR/AD	Nc	Platelet count normal or slightly decreased. Normal platelet morphology δ and α -granule defect by electron microscopy. Reduced LTA and/or absence of second aggregation wave with weak/low dose agonists (ADP, epinephrine, collagen). Defect of granular protein release by flow cytometry. Absent or very moderate hemorrhagic symptoms, associated with situations of high hemorrhagic risk.	[50,99]
α	Grey Platelet Syndrome (GPS) (139090)	AR AD	*NBEAL2* *GFI1B*	See Table 1.	
	Quebec Syndrome (QS) (601709)	AD	*PLAU*	Moderate thrombocytopenia and normal morphology. LTA absent or reduced with epinephrine and normal with other agonists. Defect of procoagulant platelet activity and increased fibrinolytic activity α-granule protein defect by flow cytometry. (P-selectin, factor V). Mucocutaneous, visceral and/or post-surgery bleeding. Response to anti-fibrinolytics but lack of response to platelet transfusions.	[100,101]
δ	Hermansky-Pudlak Syndrome (HPS) (203300, 608233, 614072, 614073, 614074, 614075, 614076, 614077, 614171, 617050, 619172)	AR	*HPS1, * *HPS2-[* *AP3B1], HPS3, * *HPS4, * *HPS5, * *HPS6, * *HPS7-[* *DTNBP1]* *HPS8-[* *BLOC1S3]* *HPS9-[* *BLOC1S] * *HPS10-[* *AP3D1], * *HPS11 [BLOC1S5]*	Normal platelet count and morphology. Selective δ-granule defect by electron microscopy. Reduced LTA and/or absence of second wave with weak/low dose agonists (ADP, epinephrine, collagen). Radio-labelled serotonin uptake, mepacrine uptake, and CD63 release defects by flow cytometry. Oculocutaneous albinism; accumulation of lipofuscin-like ceroid material in cells of the phagocytic mononuclear system; neutropenia, immunodeficiency, pulmonary fibrosis, granulomatous colitis depending on the subtype. Genotype–phenotype relationship. Mild to moderate bleeding tendency.	[50,99,102,103,104,105]
	Chediak-Higashi Syndrome (CHS) (214500)	AR	*LYST*	Normal platelet count and morphology. δ-granule defect by electron microscopy. Reduced LTA and/or absence of second aggregation wave with weak/low dose agonists (ADP, epinephrine, collagen). Impaired uptake of radioactively labeled serotonin or mepacrine, and defect in CD63 release by flow cytometry. Oculocutaneous albinism; Immunodeficiency with predisposition to recurrent infections. In 85% of cases evolution to hemophagocytic lymphohistiocytosis with an accelerated phase. A juvenile milder form can be caused by missense variants maintaining Lyst expression/function. Genotype-Phenotype relationship. Mild to moderate bleeding tendency.	[50,99,102,106]
	Griscelli Syndromes (214450, 607624, 609227)	AR	*RAB27* *MYO5A* *MLPH*	Normal platelet count and morphology. δ-granule defect as in HPS and CHS. Variable neutropenia. Albinism, silver hair, neurological defects, lymphohistiocytosis, decreased cytotoxic function of NK cells and T-lymphocytes, according to subtype.	[50,99,107]
	Delta-storage pool disease	AD/AR	-	Normal platelet numbers. Reduced second phase of aggregation with weak/low dose agonists (ADP, epinephrine, collagen). Absence of δ-granules in electron microscopy. Absent or mild bleeding tendency.	[50,99]

Inh: inheritance; AD: autosomal dominant; AR: autosomal recessive; LTA: light transmission aggregometry.

**Table 4:** Other inherited platelet disorders.

Affected Element	Disease (OMIM Code)	Inh.	Genes	Clinical & Laboratory Phenotype in Most Reported Cases	Ref.
Enzyme activity/signaling pathways	Defect in TxA2 pathways (176805, 231095, 600522)	AR/AD	*PTGS1 TBXAS1* *PLA2G4A*	Normal platelet count and morphology. Occasionally moderate thrombocytopenia. Selectively reduced LTA with arachidonic acid and normal with TxA2 analogs such as U46619. Reduced with weak/low dose agonists (ADP, epinephrine, collagen). Serum TxA2 generation defect. Heterozygous variant in PTGS1 affecting N-glycosylation may have dominant negative effect. Osteoporosis is a feature in Ghosal syndrome (*TBXAS1* defect); recurrent gastrointestinal ulceration may associate to *PLA2G4A* defect. Moderate or absent bleeding symptoms; usually associated with high bleeding risk situations.	[8,108,109,110]
	G protein defects (Gi, Gq, Gs, Gz)	-	*GNAS* *GNAQ* *GNAZ* *GNAI1*	Normal platelet count and morphology. Gs hyperfunction may be associated with discrete macrothrombocytopenia and neurological disorders. Reduced LTA and secretion in response to multiple soluble agonists. Moderate hemorrhagic symptoms; mainly associated with hemostatic challenges.	[8,111,112]
	CalDAG-GEF1 defect (615888)	AR	*RASGRP2*	Normal platelet count and morphology. LTA and secretion defects with weak agonists. αIIbβ3 integrin activation defects with weak agonists (fibrinogen binding deficit, PAC-1). Normal or moderately reduced response to PMA.	[113,114,115]
	LAD-III Syndrome (Kindlin-3 deficiency) (612840)	AR	*FERMT3*	Leukocyte adhesion deficiency syndrome type 3 (LADIII): infections, poor healing, osteopetrosis. Severe bleeding tendency, even more severe than Glanzmann thrombasthenia.	[116,117]
Procoagulant Activity	Scott Syndrome (262890)	AR	*ANO6* *[TREM16F]*	Normal platelet count and morphology. Decreased agonist-induced generation of microparticles and annexin V platelet binding, demonstrable with flow cytometry. Abnormal clot formation and retraction. Moderate to severe bleeding; mainly in risk situations (childbirth, dental interventions).	[8,118]
	Stormorken Syndrome (185070)	AD	*STIM1* *ORAI1*	See Table 1.	

Inh: inheritance; AD: autosomal dominant; AR: autosomal recessive; LTA: light transmission aggregometry; TxA_2_: Tromboxane A_2_ LAD: leucocyte adhesion deficiency.

**Table 5:** Stratification of tests in the diagnosis of inherited platelet disorders.

Value of Commonly Used Assays in the Diagnosis of IPD (0: Low; 1: Reasonable; 2: High)#	Basic Panel	Extended Panel (Confirmation/Characterization)
Complete blood counts and blood smear: 2 Bleeding time: 0 PFA-100: 1 Thromboelastography: 0 Platelet aggregation: 2 Lumiaggregometry: 2 Flow cytometry: 2 Electron microscopy: 2 Molecular analysis: 2	Complete blood counts (automatic) Blood smear (platelet count and morphology, inclusion bodies in neutrophils, abnormalities in other blood cells) Basic coagulation and VWF study Platelet aggregation: basic panel of agonists: PAR-1 peptide (TRAP), ADP, arachidonic acid, epinephrine, collagen, ristocetin. Flow cytometric analysis of major platelet adhesive glycoproteins (GP Ib/IX; GP IIb/IIIa; GP Ia/IIa, GPVI)	Immunostaining of platelet proteins in blood smears. Platelet secretion: ATP release by lumiaggregometry; HPLC; uptake/release of serotonin (HPLC/ radiolabelled); release of CD62/CD63 by flow cytometry; release of granular proteins (PF4. Thrombospondin-1, VWF, P-selectin,) by ELISA. Agonist-induced activation of αIIbβ3 by flow cytometry (Binding of PAC-1 or fibrinogen labeled with fluorochromes). Clot retraction assay. Platelet aggregation: basic panel of agonists: PAR-4, collagen related peptide [CRP], convulxin, PMA, U46619, A23188; aggregation inhibition test with PGE1 or PGI2. Extended panel of platelet receptor expression (ADP receptors, TxA_2_, thrombin; CD31) by flow cytometry with selective antibody or ligand binding assays. Procoagulant activity by cytometry (annexin V binding). Measurement of second messengers (Ca2 +, IP3, cAMP, TxA_2_) by cytometry or biochemical assays. Assessment of signaling cascades by measuring phosphorylation of specific proteins by western blot or flow cytometry (VASP test). Platelet adhesion and spreading on adhesive surfaces (static and/or low flow). Platelet morphology/ultrastructure (electron microscopy, immunofluorescence microscopy, confocal). Molecular analysis (first-line HTS with a panel of selected genes; negative cases in HTS-gene panel can be analyzed by complete whole exome sequencing [WES) and/or complete genome sequencing [WGS].

vWF: von Willebrand factor; HTS: high throughput sequencing; ADP: adenosine diphosphate; PF4: platelet factor 4; For more details see previous publications [2,130,131]; #A score of 0 means that the test add little for the diagnosis of IPD, while test scored 2 are considered as diagnostic of IPD by them self. Evaluation of blood smears with classical MGG staining is almost mandatory in the diagnosis process of IPD, as it would provide essential information in the number and morphology of platelets. Blood smear can also inform of other blood cell abnormalities that are typical of some IPDs, such as inclusion bodies in neuthrophils present in some patients with MYH9-RD. Recently, immunofluoresecence staining of platelet proteins has been shown as a helpful assay in the diagnosis of IPD [132,133]. Bleeding time is an old test measuring the time to cessation of bleeding after a standardized incision in the forearm of the subject [134]. This test was shonw to have great variability among laboratories and low sensitivity (30%) among patients with mucocutaneous bleeding history [135], and it is longer recommended by experts for the diagnosis of IPD [130]. The Platelet Function Analyzer (PFA-100^©^, Siemens) is a point-of care that automatically measures the time until occlusion of blood flow through a small orifice (150 µm diameter) in a membrane located inside a disposable cartridge, as a result of platelet adhesion and aggregation induced simultaneously by a high flow rate (4000-6000 s-1) and by a mixture of collagen with ADP or with epinephrine in the membrane The tests is highly sensitive for screening of severe IPD or VWD, but PFA-100 results can be normal in moderate disorders. Therefore, PFA-100 testing should be considered optional in the evaluation of platelet disorders [136,137]. Light transmission aggregometry, originally developed by Born, is still the gold-standard in platelet function testing and in diagnosis of IPD [138,139]. This assay, essentially measures the change in light transmission across a sample of platelet-rich-sample as platelets aggregate in response to an agonist added to the sample. Specific platelet aggregation profiles are diagnostic of certain severe IPD such as BBS or GT (see main text above). Much international effort has been done to standardize this milestone test [140]. Platelet aggregation can be combined with measurement of ATP release in lumiagregometry assays [137]. Agregation can also be mesured in whole blood by point-of-care such as Multiplate or by means of adapted flow cytometry o microfluidic assays [137]. Flow cytometry has became a routine procedure in the diagnosis of many hematological diseases including IPD. By means of fluorochrome-labelled specific antibodies or ligands specific for platelet proteins (surface receptors, granule markers, signaling proteins, etc.), flow cytometers can discriminate platelet populations with different expression of these proteins [141]. Electron microscopy allow detailed examination of platelet ultrastructure and can, therefore be diagnostic of some IPD such a GPS, a severe congenital defect of α-granules [142]. A less technical demanding procedure is whole mount electron microscopy, which allows quantification of dense granules in a non-manipulated drop of platelet rich plasma. Whole mount can be diagnostic of severe α-granules defect such as HPS [99]. Lastly, genetic diagnosis, i.e., identification of the underlying molecular defect, provides the definitive confirmation of an IPD. Until two decades ago, Sanger sequencing of candidate genes was the almost unique molecular tool, but currently HTS of panels of predefined genes, or in some cases even the whole exome o genome, have been introduced in first line genetic diagnosis of IPD [2,125,126,143].

## Data Availability

Not applicable.
